# Australian Notes

**Published:** 1911-01-21

**Authors:** 


### AUSTRALIAN NOTES.

(FROM A MELBOURNE CORRESPONDENT.

### THE HOSPITALS.

At last tenders for the construction of the first part of the new home of the Melbourne Hospital have been accepted, and work has been commenced by pulling down the Secretary's house before commencing the out-patients' department and nurses' home. Beginnings are also to be made with Block D front surgical wards and Block F front medical wards. The Medical Superintendent has submitted a proposed improvement in dealing with out-patients. At present all the honorary medical officers attend in the morning only, with resulting congestion of the waiting-rooms and some patients having to stand out in the weather. It is proposed that attendance should be made in the afternoon as well as morning.

A united movement of citizens is in progress to raise funds for the Alfred Hospital and prevent the closing of any of the wards.

The Women's Hospital has often complained of lack of public sympathy. The complaint was renewed at the last fortnightly meeting, when it was stated that the lack is still more accentuated now that life members have been deprived of their votes.

Increased accommodation is badly needed at the Melbourne Eye and Ear Hospital. For the quarter ending October 30 the number of new cases was 1,841, against 1,675 for the same period last year. On a day just before the committee meeting forty-six cases appeared, nine of which were admitted as in-patients.

A "Private Hospitals Regulation and Inspection Bill" is now before the State (Victorian) Parliament, which provides that such hospital premises shall be inspected every three months, and that the case-book shall also be open to the Public Health Board's Inspector.

The Queen Victoria Women's Hospital recently received the sum of $1,000 for the Building Fund from the trustees of the late Mr. H. A. Massey, of Toronto.

Within three days after the great "Melbourne Cup" race was won (on November 1) by "Comedy King," his owner, Mr. S. Green, distributed £500 among nine metropolitan hospitals and two charitable societies.

### LUNACY.

The Victorian Inspector-General for the Insane (Dr. Ernest Jones) is pushing on his improvements. The new establishment at Mont Park, Heidelberg, some eight miles from Melbourne, was commenced in July. It is to be on the cottage system, and some half-dozen buildings are being erected to receive the first batch of quiet male patients, who will then be put to work on the improvement of the grounds, some 1,200 acres. It will take about ten years to complete the Mont Park Asylum. It will then afford accommodation for the patients now at Yarrow Bond and in the Idiot Wards at Kew. Finally, the Kew Asylum itself may be abolished. The Sunbury Asylum is to be remodelled in the near future.

The Receiving House, also due to Dr. Jones, is found to have a good effect, as it intercepts and cures many slight cases that otherwise would have gone to the asylums. A hospital has been erected at this house, but has not yet been put into use, pending some amendments in the Lunacy Act.

Victoria still occupies the bad eminence of topping the list with 394 insane persons per 100,000 of population; Queensland shows 392, New South Wales 354, New Zealand 350, Tasmania 269, West Australia 261, and South Australia 258. Dr. Jones accounts for Victoria's high proportion as due to a larger percentage of old persons in the population and the more extensive use of the trial-leave system, which causes names to remain much longer on the registers of the Victorian Asylums than is the case in any of the other States.

The population of the hospitals for insane has decreased owing to the number now allowed out on trial-leave or boarded out with friends, guardians, or benevolent asylums.

### RADIUM IN AUSTRALIA.

Since the discoveries of radium-bearing ore, mentioned in these notes of August 31, we have heard little more upon the subject of a domestic supply of the matter until a week ago, when Dr. Douglas Mawson, lecturer on mineralogy at the University of Adelaide (South Australia), returned from a journey of exploration towards Lake Frome. The southern end of this lake is, roughly 31° south. Dr. Mawson has brought back news of an extensive radium-bearing lode in very rough country in that district. Meantime the authorities of the Sydney Hospital, where experiments have been carried out with £900 worth of radium, with very favourable results, have resolved to send an honorary surgeon of the hospital to Paris to purchase £4,000 worth of radium, disregarding Professor Anderson Stuart's protest against rushing into this expense, on the ground that radium was likely to be much cheaper ere long, and because, according to recent opinions of eminent authorities, the Röntgen rays would accomplish all that was claimed for radium.

In Melbourne the Alfred Hospital has just received the gift of £125 worth of radium from a lady, and the State Treasurer of Victoria has asked and received from the Chairman of the Board of Public Health a report on the question of a State purchase of radium for the use of the medical profession of the State. Dr. Burnet Ham's reply is wholly favourable.

